# Identifying high-confidence variants in human cytomegalovirus genomes sequenced from clinical samples

**DOI:** 10.1093/ve/veac114

**Published:** 2022-12-05

**Authors:** Salvatore Camiolo, Joseph Hughes, Fausto Baldanti, Milena Furione, Daniele Lilleri, Giuseppina Lombardi, Micol Angelini, Giuseppe Gerna, Maurizio Zavattoni, Andrew J Davison, Nicolás M Suárez

**Affiliations:** School of Infection and Immunity, MRC-University of Glasgow Centre for Virus Research, Glasgow G61 1QH, UK; School of Infection and Immunity, MRC-University of Glasgow Centre for Virus Research, Glasgow G61 1QH, UK; Department of Clinical, Surgical, Diagnostic and Pediatric Sciences, School of Infection and Immunity, University of Pavia, Pavia 27100, Italy; Microbiology and Virology Department, Fondazione Istituto di Ricovero e Cura a Carattere Scientifico (IRCCS) Policlinico San Matteo, Pavia 27100, Italy; Microbiology and Virology Department, Fondazione Istituto di Ricovero e Cura a Carattere Scientifico (IRCCS) Policlinico San Matteo, Pavia 27100, Italy; Microbiology and Virology Department, Fondazione Istituto di Ricovero e Cura a Carattere Scientifico (IRCCS) Policlinico San Matteo, Pavia 27100, Italy; Neonatal and Intensive Care Unit, Fondazione IRCCS Policlinico San Matteo, Pavia 27100, Italy; Neonatal and Intensive Care Unit, Fondazione IRCCS Policlinico San Matteo, Pavia 27100, Italy; Transplant Research Area and Centre for Inherited Cardiovascular Diseases, Fondazione IRCCS Policlinico San Matteo, Pavia 27100, Italy; Microbiology and Virology Department, Fondazione Istituto di Ricovero e Cura a Carattere Scientifico (IRCCS) Policlinico San Matteo, Pavia 27100, Italy; School of Infection and Immunity, MRC-University of Glasgow Centre for Virus Research, Glasgow G61 1QH, UK; School of Infection and Immunity, MRC-University of Glasgow Centre for Virus Research, Glasgow G61 1QH, UK

**Keywords:** human cytomegalovirus, congenital infection, intrahost evolution, sequence variability

## Abstract

Understanding the intrahost evolution of viral populations has implications in pathogenesis, diagnosis, and treatment and has recently made impressive advances from developments in high-throughput sequencing. However, the underlying analyses are very sensitive to sources of bias, error, and artefact in the data, and it is important that these are addressed adequately if robust conclusions are to be drawn. The key factors include (1) determining the number of viral strains present in the sample analysed; (2) monitoring the extent to which the data represent these strains and assessing the quality of these data; (3) dealing with the effects of cross-contamination; and (4) ensuring that the results are reproducible. We investigated these factors by generating sequence datasets, including biological and technical replicates, directly from clinical samples obtained from a small cohort of patients who had been infected congenitally with the herpesvirus human cytomegalovirus, with the aim of developing a strategy for identifying high-confidence intrahost variants. We found that such variants were few in number and typically present in low proportions and concluded that human cytomegalovirus exhibits a very low level of intrahost variability. In addition to clarifying the situation regarding human cytomegalovirus, our strategy has wider applicability to understanding the intrahost variability of other viruses.

## 1. Introduction

Human cytomegalovirus (HCMV; species *Human betaherpesvirus 5*) causes serious disease in transplant recipients and people with immune deficiencies and is a leading cause of congenital infection worldwide (Manicklal et al. 2013). Licensed vaccines are not yet available, but infections can be treated by timely therapy with antiviral drugs. However, HCMV strains are capable of generating drug-resistant variants that may become predominant and cause significant clinical problems (Lurain and Chou 2010). The inherent variability of HCMV strains that this exemplifies, in combination with the occurrence of infections involving more than one strain, creates the potential for HCMV to evolve within a patient and contribute to the wide spectrum of disease conditions associated with infection. In addition, the possibility that different variants or strains may compartmentalize (Peek et al. 1998; Tarragó et al. 2003; Miller et al. 2006) has further implications in pathogenesis, diagnosis, and treatment (Frange et al. 2013; Slyker et al. 2014), since the viruses characterised in the screened compartment may not reflect those in the organ affected. As a result, there is considerable interest in the variability and evolution of HCMV, both in the long term within human populations (interhost evolution) and in the short term within individuals (intrahost evolution). Knowledge of both aspects has been informed predominantly by high-throughput sequencing (HTS), initially of viruses isolated in cell culture and then, usually by incorporating target enrichment, directly of viruses present in clinical samples.

HCMV has a double-stranded DNA genome of approximately 236 kbp that encodes at least 170 functional proteins (Dolan et al. 2004; Gatherer et al. 2011; Davison et al. 2013). These proteins include a proofreading DNA polymerase and a suite of factors that manipulate the host immune system to facilitate lifelong infection (Van Damme and Loock 2014; Wilkinson et al. 2015). Knowledge of the interhost evolution of HCMV rests on the identification of differences between the consensus genome sequences of individual strains. Major discoveries include the presence of hypervariable genes, each of which is apparent as several distinct genotypes, the existence of huge numbers of recombinant strains presumably promoted by multiple-strain infections, and the circulation of strains containing disrupted, non-functional genes (Sijmons et al. 2015; Lassalle et al. 2016; Suárez et al. 2019a). In contrast, knowledge of the intrahost evolution of HCMV depends on the identification of strain variants, which, because they are often present in low proportions, is particularly sensitive to the limitations of HTS. Thus, although HTS has been used widely for quantifying the diversity of HCMV and other viruses in clinical samples (Beerenwinkel et al. 2012), it is important to address key technical factors in order to ensure that the variants identified are indeed due to intrastrain evolution and not artefactual (Ross et al. 2013; Sato et al. 2019). For example, the extensive use of polymerase chain reaction (PCR) makes HTS prone to generating errors (Potapov and Ong 2017) that, via the amplification of multiple identical reads from a single target genome fragment (read duplication or clonality), may result in overestimates of variability and evolutionary rate (Smith et al. 2014; Kebschull and Zador 2015). Also, low-quality sequence data and non-systematic (random) errors even in data that are of high quality overall may make intrastrain variants difficult to differentiate from artefacts. The former limitation should be controlled by screening datasets for quality and the latter by analysing datasets generated from replicate sequencing libraries (although this is rarely done). Several other sources of artefact may also be introduced as the result of additional technical factors (Dohm et al. 2008; Meacham et al. 2011; Nakamura et al. 2011).

Initial investigations of congenitally infected newborns led to the surprising conclusion that HCMV exhibits a high level of intrahost variability, thus implying that the virus evolves rapidly, at a rate similar to that of RNA viruses (Renzette et al. 2011, 2013, 2014, 2017). In contrast, subsequent work indicated that intrahost variability is much lower, as expected of a virus encoding a proofreading DNA polymerase, and that it is significantly perturbed only by the selection pressure exerted by antiviral drugs or by shifts in the relative sizes of HCMV populations in multiple-strain infections (Hage et al. 2017; Cudini et al. 2019; Suárez et al. 2019a; Suárez et al. 2020; Houldcroft et al. 2020; Jensen and Kowalik 2020). It is possible that the initial investigations were affected by the technical limitations of HTS or that some datasets represented more than one HCMV strain, either because of multiple-strain infection of the patient or as a result of cross-contamination events. Both situations would lead to overestimates of the rate of intrahost evolution, the first because of the contribution of artefacts and the second because the variability detected would reflect interhost, rather than intrahost, diversity.

The approaches taken to date in studies of the intrahost evolution of HCMV have differed considerably, and their limitations have been managed with varying degrees of attention. In our study, we developed a stringent approach for identifying HCMV variants in clinical samples. We established this approach using sequence data (including biological and technical replicates) generated from samples collected longitudinally from different compartments (blood, saliva, and urine) from a cohort of three congenitally infected newborns. We also re-examined selected published sequence data. In addition to providing a means of guiding future studies, our findings confirm the view that reproducible, high-confidence intrahost HCMV variants are rare and generally present at low levels and therefore that the intrahost evolution of HCMV is slow.

## 2. Materials and methods

### 2.1 Samples

**Figure 1.**
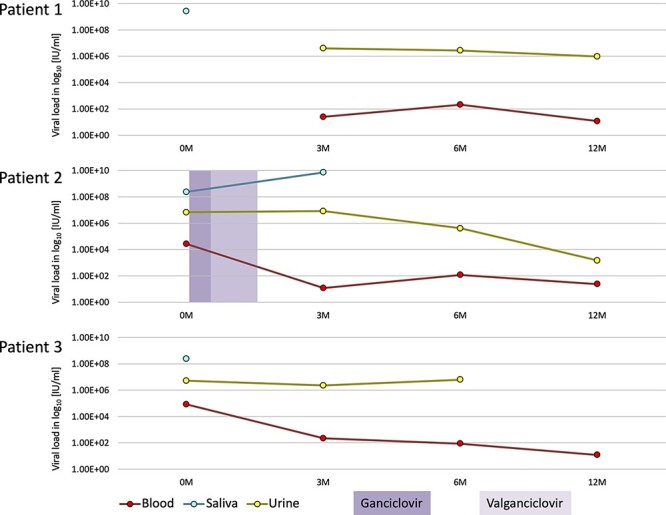
Viral population dynamics of the cohort samples. As indicated in the key, the colours of the lines represent the sample types, and the duration of antiviral therapy is represented by the coloured blocks. Approximate sampling time points (M, months) are indicated on the *x* axis and viral loads (log_10_ IU/mL) on the *y* axis.

A total of 33 clinical samples (whole blood, *n* = 11; saliva, *n* = 12; and urine, *n* = 10) were collected longitudinally from a cohort of three confirmed HCMV-infected newborns (patients 1, 2, and 3) at four time points that corresponded approximately to the first 3 weeks (0 M), 3 months (3 M), 6 months (6 M), and 12 months (12 M) of life. Viral loads in most samples (*n* = 25) were measured at the collection site as described previously (Gerna et al. 2006). The titres ranged from <1.2 × 10^1^ (the lowest level detectable) to 8.4 × 10^4^ HCMV international units (IU)/ml in whole blood, 2.4 × 10^8^–7.2 × 10^9^ IU/ml in saliva, and 1.5 × 10^3^–8.4 × 10^6^ IU/ml in urine (Table 1 and Fig. 1). Patient 2 underwent antiviral therapy with ganciclovir (6–16 days after birth) and valganciclovir (17–46 days after birth). Samples were collected with the approval of the institutional review boards of Policlinico San Matteo, Pavia (reference number 5908/2014).

**Table 1.** Clinical information on the cohort.

				Sample collection		
Patient	Clinical findings at birth	Antiviral regime	Clinical findings at follow-up	Time point	Compartment	Viral load (IU/mL)
1	Poor motility, germinolysis	None	No symptoms	0 M	Saliva	2.6 × 10^9^
				3 M	Blood^a^	2.5 × 10^1^
					Saliva	NE
					Urine	4.0 × 10^6^
				6 M	Blood^a^	2.1 × 10^2^
					Saliva	NE
					Urine	2.8 × 10^6^
				12 M	Blood^a^	1.2 × 10^1^
					Saliva	NE
					Urine	9.6 × 10^5^
2	IUGR, low platelet count, hepatitis, poor motility	GCV, vGCV	SNHL, hypotonia, tremors	0 M	Blood	2.7 × 10^4^
					Saliva	2.4 × 10^8^
					Urine	6.9 × 10^6^
				3 M	Blood^a^	1.2 × 10^1^
					Saliva	7.2 × 10^9^
					Urine	8.4 × 10^6^
				6 M	Blood^a^	1.2 × 10^2^
					Saliva	NE
					Urine	4.1 × 10^5^
				12 M	Blood^a^	2.4 × 10^1^
					Saliva	NE
					Urine	1.5 × 10^3^
3	Asymptomatic	None	No symptoms	0 M	Blood	8.4 × 10^4^
					Saliva	2.4 × 10^8^
					Urine	5.2 × 10^6^
				3 M	Blood	2.2 × 10^2^
					Saliva	NE
					Urine	2.3 × 10^6^
				6 M	Blood^a^	8.7 × 10^1^
					Saliva	NE
					Urine	6.2 × 10^6^
				12 M	Blood^a^	1.2 × 10^1^
					Saliva^a^	NE

a Sequencing library not generated.

Abbreviations: 0 M, within 3 weeks; 3 M, 3 months; 6 M, 6 months; 12 M, 12 months; GCV, ganciclovir; vGCV, valganciclovir; IUGR, intrauterine growth restriction; SNHL, sensorineural hearing loss; NE, viral load not estimated.

### 2.2 Sequencing libraries and sequencing

DNA was extracted from 140 to 200 μl of sample using a QIAamp DNA blood mini kit for whole blood samples, a QIAamp MinElute virus spin kit for saliva samples, and a QIAamp viral RNA mini kit for urine samples (QIAGEN, Crawley, UK). A 50 μl aliquot of DNA was sheared acoustically using an LE220 sonicator (Covaris, Woburn, MA), aiming to achieve an average fragment size of 500 bp. The fragmented DNA was prepared for sequencing using a KAPA LTP library preparation kit (KAPA Biosystems, London, UK), incorporating the SureSelect^XT^ v1.7 target enrichment system (Agilent Technologies, Santa Clara, CA) with a custom RNA bait library as described previously (Hage et al. 2017; Suárez et al. 2019a). The libraries were single-indexed (with indexes mimicking the standard Illumina set [Supplementary Table S1]) using ultrapure (TruGrade) oligonucleotides (Integrated DNA Technologies, Leuven, Belgium) and analysed using a MiSeq DNA sequencer (Illumina, San Diego, CA) with a v3 reagent kit to generate datasets consisting of 2 × 300 nucleotide (nt) paired-end reads.

**Figure 2.**
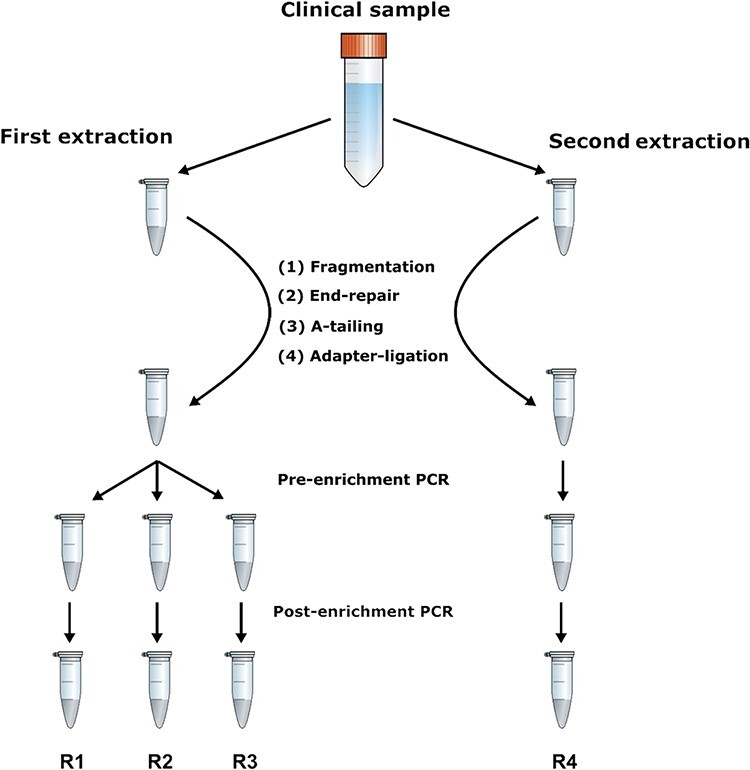
Schematic representation of the experimental design for generating biological and technical replicate sequencing libraries.

Matching saliva and urine samples collected from patient 2 at time point 3 M and patient 3 at time point 0 M were sequenced as replicates (Fig. 2). Briefly, DNA was extracted from two aliquots of each sample (biological replicates), and the initial sequencing library prepared from the first extraction was split into three technical replicates prior to pre-enrichment PCR amplification, thus leading to three sequencing libraries (R1–R3). Another sequencing library (R4) was produced from the second extraction.

The datasets were coded as follows: (1) the compartment (B, blood; S, saliva; and U, urine), (2) the patient number (1–3), (3) the time point at which the sample was collected (0 M, 3 M, 6 M, or 12 M), and when appropriate, (4) the replicate designation (R1–R4). For example, the code S2_3 M_R1 represents the first replicate of the saliva sample collected from patient 2 at approximately 3 months old. The sequencing libraries were sequenced in four separate runs. Each run contained HCMV sequencing libraries belonging to other studies, and these were coded with the prefix X and a consecutive number. Sequencing libraries were prepared in batches of 16 using two 8-well tube strips (Supplementary Fig. S1). Hypervariable HCMV genes were genotyped on the basis of enumerating reads containing conserved genotype-specific motifs, employing several motifs per genotype, using GRACy v0.4.4 (Camiolo et al. 2021).

### 2.3 Genome sequences

Sequence assemblies were generated as described previously (Suárez et al. 2019a). Briefly, a dataset was prepared for analysis using Trim Galore v0.4.0 (http://www.bioinformatics.babraham.ac.uk/projects/trim_galore/; length = 21, quality = 10, and stringency = 3). The trimmed dataset was aligned to the UCSC hg19 human reference genome sequence (http://genome.ucsc.edu/) using Bowtie2 v2.3.1 (Langmead and Salzberg 2012). Nonmatching reads were assembled *de novo* into contigs using SPAdes v3.5.0 (Bankevich et al. 2012) (—careful, which reduces the number of mismatches and short indels in the contigs generated—k 21,33,55,77,99,127). The contigs were organised using Scaffold_builder v2.2 (Silva et al. 2013), employing as a reference the genome sequence of HCMV strain Merlin (GenBank accession number AY446894.2) with all but 100 nt of the terminal repeat regions removed. An alignment of the dataset to the assembled genome made using Bowtie2 was inspected using Tablet v1.21.02.08 (Milne et al. 2013), and final adjustments were made to generate the genome sequence, which was annotated using GRACy.

Sequencing libraries belonging to other studies that were included in the runs were also genotyped using GRACy. Datasets representing multiple HCMV strains were assembled into sets of contigs, rather than complete genomes, using SPAdes (—careful—cov-cutoff auto).

### 2.4 Preprocessing 

**Figure 3.**
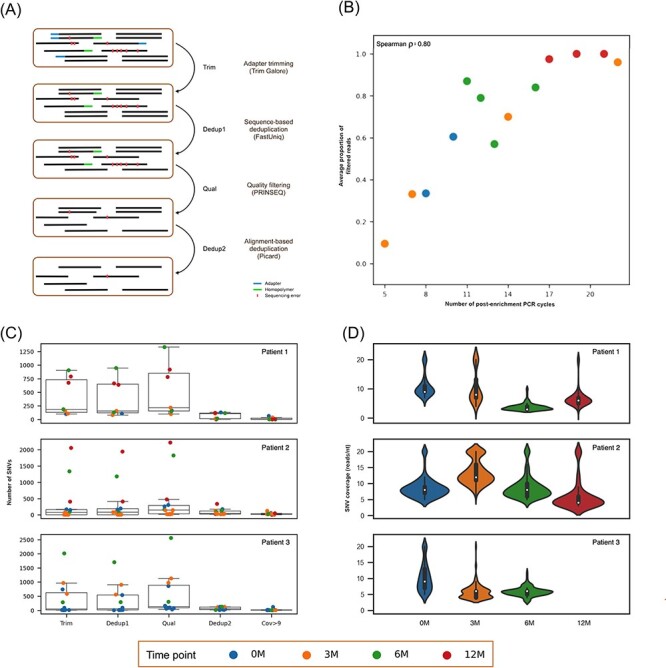
Application of preprocessing steps to the cohort datasets. (A) Schematic representation of the filtering steps (see text for details), in which each line represents a paired-end read. (B) Correlation between the proportion of reads removed and the number of PCR cycles performed during the post-enrichment amplification step. The proportions of reads removed for sequencing libraries undergoing the same number of PCR cycles were averaged. (C) Number of SNVs detected using LoFreq after each filtering step and the number remaining after application of the Cov > 9 threshold. (D) Violin plots showing the distribution of the number of reads identifying the SNVs detected using LoFreq after applying the filtering steps at each time point. The number of supporting reads was set to 20 when >20.

The original datasets were depleted of human reads as described above and then preprocessed by four sequential filtering steps(Fig. 3A). In the first step (Trim), adapters, poor-quality sequences, and short sequences were removed using Trim Galore with default parameters. In the second step (Dedup1), read clonality arising during the pre- and post-enrichment PCR amplification steps was minimized by deduplication using FastUniq v1.1 (Xu et al. 2012), on the basis of read pairs having identical sequences. In the third step (Qual), a stringent quality filter was applied using PRINSEQ v0.20.4 (Schmieder and Edwards 2011) with the following criteria: (1) reads were retained if their average quality score was >25, (2) the 3′ end of each read was trimmed if the mean quality score was <30, using a sliding window (—trim_qual_window) of 5 nt and a step size (—trim_qual_step) of 1 nt, (3) homopolymeric sequences of >20 nt were trimmed from the 3ʹ end of reads, and (4) only reads with a residual length of ≥80 nt were retained. Thus, this step was not limited to the removal of low-quality sequences but also involved the removal of artefactual homopolymeric G-tracts with high-quality values that have been reported to occur at the 3ʹ end of reads generated on the Illumina platform (https://sequencing.qcfail.com/articles/illumina-2-colour-chemistry-can-overcall-high-confidence-g-bases/). In the fourth step (Dedup2), read clonality was minimized by deduplication using Picard (http://broadinstitute.github.io/picard/) on the basis of read pairs having identical mapping coordinates. For this, reads were aligned to the relevant genome sequence using Bowtie2 (—end-to-end). The resulting SAM file was converted to BAM format and sorted and indexed using Samtools v1.3 (Li et al. 2009).

### 2.5 Variant detection

Single nucleotide variants (SNVs) present in minor genome populations were identified in the preprocessed datasets using two programs: (1) LoFreq v2.1.5 (Wilm et al. 2012) (-Q 30, so that nucleotides calling the reference nucleotide were considered only if their quality was ≥30; -q 30, so that positions at which an alternative nucleotide was called were considered only if their quality was ≥30) and (2) VarDict v1.8.2 (Lai et al. 2016) (-q 30, so that only nucleotides with a quality of ≥30 were considered). LoFreq implements a quality-filtering step on SNVs by default (minimum variant calling quality score = 58, minimum coverage = 10, and a strand bias false discovery rate correction *P* value of >0.001). VarDict reports all SNVs without additional filtering (although several downstream filtering options are proposed in the software documentation). Since HTS can lead to errors associated with read directionality (Stoler and Nekrutenko 2021), only SNVs confirmed by at least two reads in each direction were included, thus requiring a coverage depth of ≥4 reads/nt.

### 2.6 Detection of variants due to cross-contamination

For each SNV, reads calling the alternative nucleotide were identified using Sam2Tsv (http://dx.doi.org/10.6084/m9.figshare.1425030). Reads presenting >1 mismatch to the consensus were aligned using Bowtie2 (with default parameters) to all other genome sequences (complete or partial) assembled from samples sequenced in the same run. Reads aligning without mismatches to any of the other sequences were considered to have arisen from contamination, on the basis that they were unlikely to have arisen by error. The number of contaminating reads was computed for each SNV in this category.

### 2.7 Reproducible detection of variants

Variants detected in at least three of the four replicate datasets from patient 2 (S2_3 M and U2_3 M) and patient 3 (S3_0 M and U3_0 M) were categorised as reproducible SNVs. This threshold allowed a reproducible SNV to be absent from one replicate dataset due to low sequencing coverage or to the possibility that the same pool of SNVs was not sequenced in all replicates of samples with high viral loads.

### 2.8 Analysis of public datasets

Ten public Illumina datasets also generated by target enrichment of HCMV from clinical samples in three separate studies were processed as described above (Supplementary Table S2). These datasets were selected on the basis of exhibiting high intrahost variability of apparent single-strain infections in the corresponding studies.

## 3. Results

### 3.1 Sequencing libraries and sequencing

A total of 33 longitudinal blood, saliva, and urine samples were obtained from a cohort of three congenitally HCMV-infected newborns, of whom two (patients 1 and 2) were symptomatic and one (patient 3) was asymptomatic (Table 1). Sequencing libraries were produced for the 24 samples that had sufficient viral loads. Saliva samples had the highest viral loads, followed by urine and then blood samples (Fig. 1). Patient 2 presented the highest viral load at time point 3 M in saliva, despite having undergone antiviral therapy during the first month. However, the viral load in blood samples from this patient decreased from 0 M to 3 M. The symptomatic patients presented higher viral loads in saliva samples (S1_0 M = 2.6 × 10^9^ IU/mL and S2_3 M = 7.2 × 10^9^ IU/mL) than the asymptomatic patient (S3_0 M = 2.4 × 10^8^ IU/mL).

In addition, 12 replicate sequencing libraries were produced from matched saliva and urine samples from patients 2 and 3, bringing the total number of sequencing libraries analysed to 36. Information on the sequencing libraries and resulting datasets is provided in Supplementary Table S3. There were 21,306–4,316,790 trimmed reads per dataset, with 27–93 per cent mapping to the HCMV strain Merlin reference genome at an average coverage depth of 6–3,920 reads/nt.

Genotyping of 13 hypervariable genes for the 36 datasets demonstrated that each of the three patients was infected by a single HCMV strain (Supplementary Fig. S1). This was in contrast to several of the datasets (prefixed by X in Supplementary Fig. S1) that were included in the sequencing runs but belonged to other studies, for which the detection of additional genotypes indicated that multiple strains were present.

### 3.2 Genome sequences

The complete HCMV genome sequences for patients 1, 2, and 3 were determined from datasets S1_0 M, S2_6 M, and U3_6 M and had sizes of 235,481, 235,825, and 235,405 bp, respectively. These sequences were used as references to assess variability as described below. Two genes were disrupted in the genome sequences (Supplementary Fig. S2), namely UL9 (encoding a hypervariable membrane glycoprotein) in patient 1 and UL111A (encoding an interleukin-10 homologue) in patients 2 and 3. Disruptive mutations causing premature translational termination in these two genes have been reported previously in clinical HCMV strains, and the precise disruption in patient 3 has been identified in several other strains (Suárez et al. 2019a).

### 3.3 Preprocessing

The datasets generated from the three blood samples that had sufficient viral loads were relatively small, and one of these (B3_3 M) presented with low coverage depth and high read clonality (Supplementary Table S3). As a result, all datasets generated from blood samples were excluded from variant detection, and only 33 datasets (including the 12 replicates), all derived from saliva and urine samples, were processed.

To reduce the impact of biases and sources of error inherent in the sequence data, four filtering steps were applied sequentially to the datasets after depleting human reads (Fig. 3A): Trim, which removes adapters and poor-quality sequences; Dedup1, which removes clonal reads on the basis of sequence identity; Qual, which stringently removes poor-quality sequences; and Dedup2, which removes duplicate read pairs on the basis of mapping location. The order of these steps and the parameters used in each were determined on the basis of extensive experimentation. The effects on read numbers are summarised in Supplementary Table S4. The Trim filter removed a small proportion (<0.02 per cent) of reads from each dataset, the Dedup1 filter removed up to 34.12 per cent of reads, the proportion correlating strongly with the number of post-enrichment PCR amplification cycles (Spearman *r* = 0.8, *P* < 0.001; Fig. 3B), the Qual filter removed up to 10.77 per cent of reads and resulted in many fewer mismatches and much higher mapping quality scores (Supplementary Fig. S3), and the Dedup2 filter removed up to 99.80 per cent of reads. The final average coverage depth varied widely (2.73–3,047.13 reads/nt).

### 3.4 Variant detection

Two widely used programs (LoFreq and VarDict) were used for SNV detection because they may be performed at various levels of accuracy and sensitivity. LoFreq uses a conservative approach and has been reported to outperform other tools in terms of accuracy when analysing large DNA viruses (Deng et al. 2020), whereas VarDict is more sensitive (Bian et al. 2018) (Supplementary Table S5). The effects on SNV numbers are summarised in Supplementary Table S6 and Fig. 3C. The Qual filter tended to increase the number of SNVs identified by both programs, presumably because of its effects on the number of mismatches and the average mapping quality scores. The overall impact of the four filtering steps varied widely from increasing the number of SNVs several-fold to reducing it to zero.

### 3.5 Detection of variants due to cross-contamination

Preprocessing lends greater confidence to variant detection but does not indicate whether variants are due to intrastrain or interstrain variability. Genotyping is capable of identifying interstrain variation due to the presence of multiple HCMV strains but does not indicate whether variants are due to multiple-strain infection or cross-contamination. However, variants due to cross-contamination during a run may be identified by taking into account the sequences of the other samples analysed in the same run. This was done by identifying reads that contain >1 SNV and match exactly the consensus HCMV sequence from one of the other samples included in the same run. Among the datasets from patients 1–3, >50 per cent of reads supporting 11 per cent (*n* = 275) of SNVs identified using LoFreq after the Dedup2 filter (*n* = 2,432) were in this category. Also, although SNV detection was affected by the coverage depth at the corresponding site (Supplementary Table S6 and Fig. S4), a high proportion of SNVs (95 per cent) was supported by only a small number of reads (≤9). This threshold (Cov > 9) was then applied to all datasets, resulting in the exclusion of SNVs supported by ≤9 reads as being potentially due to contamination and in the discounting of 1,656 (68.09 per cent) and 76,237 (96.45 per cent) low-abundance SNVs identified by LoFreq and VarDict, respectively (Supplementary Table S6 and Fig. 3C). After preprocessing and applying the Cov > 9 threshold to the SNVs detected by LoFreq, a total of 96 (Supplementary Table S7), 364 (Supplementary Table S8), and 180 (Supplementary Table S9) SNVs were detected in the datasets from patients 1, 2, and 3, respectively, with no major longitudinal changes in frequency. The corresponding numbers of SNVs detected by VarDict were greater, at 229, 1,762, and 810, respectively (Supplementary Table S6).

### 3.6 Reproducible detection of SNVs

For the saliva and urine samples from patients 2 and 3 for which four replicate sequencing libraries were analysed, reproducible SNVs were defined on the basis of their detection in at least three datasets. In patient 2 and 3, respectively, those detected by LoFreq numbered 9 and 3 (Table 2), those detected by VarDict numbered 20 and 5 (Table 3), and those detected by both programs numbered 7 and 2. All SNVs were present in low proportions (≤2.2 per cent, with many at <1 per cent, especially using VarDict), with the exception of two (location 94,382 in patient 2 and location 94,348 in patient 3) that are effectively the same SNV (i.e., they are in the same sequence context but differ in relative location because the genome sequences are different) and were detected at approximately 5 per cent by LoFreq but not detected by VarDict. Five SNVs (3 in patient 2 and 2 in patient 3) were detected in both compartments, and the remaining seven were identified in a single compartment. Nonsynonymous substitutions were detected by both programs in three genes in patient 2 (UL49, UL80, and UL97) and one gene in patient 3 (UL10). The substitution (A58T) in gene UL97, in which antiviral resistance may occur, was detected in the only patient receiving antiviral therapy (patient 2).

**Table 2.** Proportions of SNVs detected in replicate datasets using LoFreq.

Patient	Location^a^	SNVs in saliva dataset (%)				SNVs in urine dataset (%)				Effects of SNV	
		S2_3M_R1	S2_3M_R2	S2_3M_R3	S2_3M_R4	U2_3M_R1	U2_3M_R2	U2_3M_R3	U2_3M_R4	Gene	Coding change
2	*72,388*	–^b^	–	–	–	0.6	0.5	0.4	1.0	UL49	L339Q
	87,491*	–	–	0.6	–	0.5	0.4	–	0.8	Intergenic	None
	94,382*	6.0	7.3	5.3	–	5.3	5.7	5.8	–	Intergenic	None
	99,587*	0.7	0.9	0.9	0.8	1.1	0.7	1.1	1.5	UL69	None
	*117,428*	–	–	–	–	0.7	0.5	0.4	–	UL80	P327T
	141,655	–	–	–	–	1.0	1.0	1.2	–	Intergenic^c^	None
	*141,906*	2.2	1.5	1.5	1.7	–	–	–	–	UL97	A58T
	*184,947*	–	–	–	–	1.6	0.8	1.1	1.8	UL141	None
	*210,231*	0.5	–	1.1	0.6	–	–	–	–	US15	None
		S3_0M_R1	S3_0M_R2	S3_0M_R3	S3_0M_R4	U3_0M_R1	U3_0M_R2	U3_0M_R3	U3_0M_R4		
3	*17,725*	0.8	1.0	1.1	–	–	–	–	–	UL10	M1I^d^
	41,993*	0.9	0.9	1.2	–	–	–	–	0.8	UL32	None
	94,348*	5.2	–	–	–	4.6	3.3	4.9	5.6	Intergenic	None

a In the corresponding HCMV genome, SNVs detected in both the saliva and urine datasets are marked by asterisks; SNVs also detected using VarDict are in italic font (see Table 3).

b Absent.

c 85 nt upstream of gene UL97.

d Change in initiation codon.

**Table 3.** Proportions of SNVs detected in replicate datasets using VarDict.

Patient	Location^a^	SNVs in saliva dataset (%)				SNVs in urine dataset (%)				Effects of SNV	
		S2_3M_R1	S2_3M_R2	S2_3M_R3	S2_3M_R4	U2_3M_R1	U2_3M_R2	U2_3M_R3	U2_3M_R4	Gene	Coding change
2	28,992	0.5	–^b^	0.5	0.4	–	–	–	–	UL23	None
	30,213	–	–	–	–	–	0.3	0.2	0.5	UL24	None
	36,565	–	–	–	–	0.3	–	0.2	0.5	UL29	None
	39,277	–	–	–	–	0.2	0.2	0.3	–	UL31	None
	65,231^*^	0.5	0.4	–	0.3	0.6	0.4	–	–	UL48	L201P
	*72,388*	–	–	–	–	0.6	0.6	0.4	0.9	UL49	L339Q
	73,852	–	–	–	–	0.3	0.2	0.2	–	UL50	None
	87,491*	–	0.4	0.5	–	0.6	0.6	0.4	0.9	Intergenic	None
	99,587*	0.9	1.1	0.9	1.1	1.0	0.8	1.2	1.2	UL69	None
	103,608	–	–	–	–	0.3	0.3	0.2	–	UL70	R350H
	111,480	0.4	0.4	–	0.5	–	–	–	–	Intergenic	None
	*117,428*	–	–	–	–	0.8	0.6	0.4	0.4	UL80	P327T
	128,505	0.4	0.3	–	0.4	–	–	–	–	UL86	None
	*141,906*	2.2	1.6	1.8	1.9	–	–	–	–	UL97	A58T
	149,661*	0.6	0.4	–	0.4	0.3	0.3	0.2	–	UL102	None
	154,724*	0.3	–	0.3	0.5	–	0.4	0.4	0.5	UL105	None
	*184,947*	–	–	–	–	1.6	1.3	1.4	1.6	UL141	None
	202,826*	0.5	0.4	–	0.8	–	1.4	0.9	–	US7	S128P
	210,231*	0.5	0.4	1.1	0.7	0.4	–	–	–	US15	None
	226,566	0.5	–	0.6	0.4	–	–	–	–	Intergenic	None
		S3_0M_R1	S3_0M_R2	S3_0M_R3	S3_0M_R4	U3_0M_R1	U3_0M_R2	U3_0M_R3	U3_0M_R4	Gene	Coding change
3	*17,725*	0.7	1.4	1.0	–	–	–	–	–	UL10	M1I^c^
	41,993*	0.8	0.8	1.1	–	–	–	–	0.7	UL32	None
	87,448	–	–	–	–	0.5	0.6	0.5	0.6	Intergenic	None
	99,554*	0.9	–	–	–	0.9	–	1.0	0.4	UL69	None
	190,102*	0.7	–	0.7	–	0.8	0.6	–	0.9	UL133	V152A

a In the corresponding HCMV genome, SNVs detected in both the saliva and urine datasets are marked by asterisks; SNVs also detected using LoFreq are in italics font (see Table 2).

b Absent.

c Change in initiation codon.

### 3.7 Analysis of public datasets

**Figure 4.**
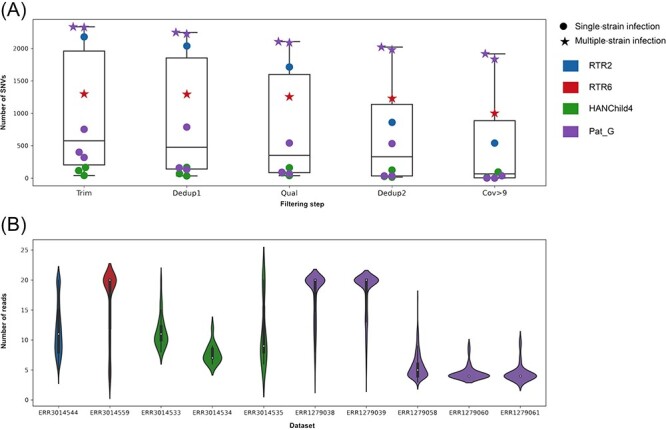
Application of dataset filtering steps to the public datasets (Supplementary Table S2). (A) Number of SNVs detected using LoFreq grouped by patient after each step. The colour of each point indicates the patient, and the shape indicates whether the dataset represented a single-strain (dot) or a multiple-strain (star) infection. (B) Violin plots showing the distribution of the number of reads identifying the SNVs detected using LoFreq for each dataset. The number of supporting reads was truncated to 25 when >20.

Ten public datasets (Supplementary Table S2) were also subjected to preprocessing and SNV detection. Preprocessing led to a reduction in the number of reads that was particularly marked after the Dedup1 and Dedup2 steps (Supplementary Table S10), amounting up to 62.17 and 69.39 per cent of reads, respectively. Preprocessing also resulted in a decrease, in some instances dramatic, in the number of SNVs detected (Supplementary Table S11 and Fig. 4A). Application of the Cov > 9 filtering step resulted in almost complete depletion of SNVs (>99 per cent) for several datasets. Although these final low numbers were in line with those registered for our cohort, some datasets retained a high number of SNVs (≥1,000), which were supported by more than 15 reads (Fig. 4B), and presented the lowest average coverage depth of SNVs (≤51 reads/nt), thus indicating the presence of additional HCMV strains in the relevant samples (Supplementary Fig. S5).

## 4. Discussion

Understanding the degree of genetic variability, and therefore the rate of intrahost evolution, of viruses is important for developing and implementing efficient strategies for management and therapy. HTS has afforded the opportunity to investigate this subject in an unprecedented level of detail. However, in order to arrive at sound conclusions, it is necessary to address the sources of bias and error that operate during the derivation and analysis of sequence data. These fall into four broad areas: (1) the presence of multiple viral strains represented in a dataset; (2) the extent to which a dataset represents the viral genome and the quality of the reads that it contains; (3) the identification of reads likely to be due to cross-contamination; and (4) the degree to which variant detection is reproducible. The varying levels of attention that these factors have received in studies of the intrahost evolution of HCMV to date prompted us to take a structured approach, applying it initially to the datasets from a cohort of three congenitally infected patients. First, we determined how many HCMV strains were represented in each patient. Second, we used read preprocessing to improve the datasets. Third, we defined a threshold for ruling out variants likely to be due to cross-contamination. Fourth, we used data from replicate experiments to identify reproducible variants in two of the patients. Fifth, we applied our approach to a selection of public datasets.

We commenced our study by using genotyping to show the presence of a single strain in each cohort patient, thus obviating all but low-level multiple-strain infection or cross-contamination as a confounding factor representing interhost variability in work aimed at investigating intrahost variability. Determining how many strains are represented in an HCMV dataset principally involves the genotyping of a selection of hypervariable genes by enumerating short genotype-specific motifs in the datasets. Methods were developed initially for detecting a single, distinct motif for each genotype (Suárez et al. 2019a,b) and were then extended in GRACy for detecting multiple motifs for each genotype, thus enhancing sensitivity (enabling the detection of minor strains present at 2 per cent) and specificity (Camiolo et al. 2021). It is likely that methods for enumerating HCMV strains will continue to improve, perhaps involving the genotyping of a larger range of hypervariable genes and the determination of the genome sequences of individual strains present in sufficient proportions (Goldstein and Wagers 2018; Cudini et al. 2019). The unrecognised presence of multiple strains in initial studies of HCMV variability may not have adequately distinguished intrastrain variability from interstrain variability, thereby contributing to the conclusion that HCMV evolves rapidly within infected individuals. Indeed, the large number of SNVs detected in these studies corresponds well with the average number of nucleotide differences (6,296) existing between pairwise interstrain comparisons of randomly selected public HCMV genome sequences (Supplementary Fig. S6 and Table S12).

We continued our study by using preprocessing to improve the quality of the datasets by four sequential read filtering steps. Two of these steps (Trim and Qual) involved the quality filtering of reads, which is important in the reliable detection of viral variants (Pfeifer 2017). The other two steps (Dedup1 and Dedup2) involved reducing read clonality. We then employed two widely used programs (LoFreq and VarDict) to detect SNVs. Although most programs for this purpose exclude low-quality data, it is important to understand their default settings. The more accurate program (LoFreq) subjects the reads to a default quality-filtering step and only considers nucleotides with Phred quality scores of >20. This corresponds to a 1 per cent probability that a variant resulted from a sequencing error (Ewing and Green 1998). However, this setting may be problematic when coverage depth is high (e.g. >1,000 reads/nt) because the probability of reporting errors as SNVs is enhanced. Indeed, previous studies have demonstrated the challenges of distinguishing genuine SNVs present in proportions of <1 per cent from errors occurring during PCR amplification or sequencing when coverage depth is high (Orton et al. 2015; McCrone, Lauring, and Dermody 2016). To reduce this problem, we considered only Phred quality scores of >30, as suggested previously (McCrone, Lauring, and Dermody 2016). The more sensitive program (VarDict) reports all SNVs without a quality-filtering step, although we implemented additional thresholds for read directionality and coverage depth. As anticipated, VarDict reported more SNVs than LoFreq.

Next, we developed a threshold from the cohort datasets for excluding SNVs likely to be due to low-level cross-contamination during the runs. Cross-contamination occurs commonly during HTS and, if present at a low level, may escape detection by genotyping. This was exemplified in the cohort by the detection of low-level cross-contamination in two datasets but not in datasets from replicate sequencing libraries (Supplementary Fig. S1; compare run 4 tubes 5 and 6 [contamination] with run 3 tubes 1 and 2 [no contamination]). Low-level SNVs may also be due to multiple-strain infection rather than cross-contamination. For example, public dataset ERR3014559 was classified initially (Suárez et al. 2019a), and by our genotyping analysis, as representing a single-strain infection (Supplementary Fig. S5) but exhibited a relatively large number of SNVs. To investigate further, we extracted the reads supporting the SNVs and searched for identity with datasets generated from the same patient at different time points that were classified as multiple-strain infections (ERR3014557 and ERR3014558; Suárez et al. 2019a). These reads showed perfect matches with reads from the additional datasets, thus suggesting that a minor strain was represented in all three datasets but in a too low proportion in dataset ERR3014559 to be detected by genotyping. We used a single threshold (Cov > 9) for all the cohort and public datasets but recognise that it may be more appropriate to calculate the threshold for future projects rather than generalising it. Also, our impression was that cross-contamination was more likely to be detected in datasets from samples with a low viral load, which require a higher number of PCR cycles during sequencing library preparation, thus increasing the probability of amplifying contaminating HCMV fragments. This suggests that a threshold determined across a cohort may be too low for such datasets. In addition, although random barcodes (unique molecular identifiers) or unique dual indexes were not used to generate the sequencing libraries, they may assist in detecting within-run cross-contamination in future studies.

Finally, we used replicate sequencing libraries to define reproducible SNVs for cohort patients 2 and 3. These SNVs were rare and present at low levels, with only seven detected in patient 2 and two in patient 3. The observation that some were detected in datasets generated from either the saliva or urine sample, but not both, may indicate compartmentalization, but the low level of occurrence indicates that this conclusion should be drawn with caution. The detection of an SNV in gene UL97 in patient 2, who underwent antiviral drug therapy, was provocative, but this SNV was identified in samples collected long after antiviral therapy had been discontinued at 46 days after birth and remained detectable at 6 months of age. In addition, it is not specifically known to cause resistance. The application of preprocessing and the Cov > 9 threshold to the public datasets had marked effects. For example, a large proportion (approximately 50 per cent) of the SNVs reported in public dataset ERR3014544 (Suárez et al. 2019a) turned out to have been derived from duplicate reads. Similarly, a marked reduction was evident in the number of SNVs reported in public datasets ERR1279060 and ERR1279061 (Houldcroft et al. 2016), including known or potential antiviral resistance mutations. However, the reported antiviral drug resistance mutations were confirmed in other samples from this patient, with the exception of one novel mutation (E235K in gene UL54). This SNV was likely to be due to a PCR error, as it was present in only a single dataset from this patient (ERR1279058), in which it was supported only by 14 clonal reads. In addition, this SNV corresponded to a C/T transition, which is the most frequent systematic error of the DNA polymerase used commonly to generate sequencing libraries (Potapov and Ong 2017).

Our study involved applying quality filters and thresholds to our cohort and public datasets, in order to detect HCMV intrastrain variants with relatively high confidence and a degree of reproducibility. However, as is always the case in such studies, this analysis involved the choice of tools and parameters at each step and could not guarantee an absolute discrimination between intrastrain variants and artefacts. Indeed, it is probable that some of the high-confidence SNVs were artefactual and some of the SNVs categorised as artefactual represent genuine intrastrain variants. For example, certain trinucleotide sequences are associated with systematic (nonrandom) sequencing errors on the Illumina platform (Schirmer et al. 2016; Stoler and Nekrutenko 2021). Although such errors are directional and are generally managed by most variant detectors by controlling strand bias, it is possible that the motifs will occur on both strands in some locations, thus creating contexts that are susceptible to high error rates (Ma et al. 2019). For example, two SNVs detected in patients 2 and 3 at locations 99,587 and 99,554, respectively, represent the same variant, which is located on both strands in association with motifs that are among those overrepresented at error sites.

## 5. Conclusion

The potential sources of bias, error, and artefact in HTS data should be addressed adequately if robust conclusions are to be drawn about the intrahost genetic diversity of viruses. In the absence of careful scrutiny, the results of such studies, while provocative, may be of unresolved scientific merit. We assessed a range of the factors involved by using clinical samples obtained from a cohort of congenitally HCMV-infected patients to develop an approach for identifying intrahost variants of HCMV. We found that the variants detected were few in number and commonly present in low proportions and concluded that HCMV exhibits a very low level of detectable intrahost variability. These findings should be set within the limitations of the methods used, which are unlikely to detect variants that arise during HCMV replication unless they are represented in a sufficient proportion of the viral population, either because they are selected and out-compete the parental strain (e.g., antiviral drug resistance mutants) or because they benefit from stochastic events that are not necessarily related to their ability to out-compete (e.g., replication bottlenecks).

## Supplementary Material

veac114_Supp

## Data Availability

The datasets generated for this study were deposited in the European Nucleotide Archive (ENA; project no. PRJEB48078), and the annotated consensus genome sequences were deposited in ENA (accession nos. ERZ4195000, ERZ4195185, and ERZ4195400). A conda environment with all the tools and resources used in the pipeline is available (https://github.com/josephhughes/HCMVmut).
