# Supplementary figures and images for: Identifying high-confidence variants in human cytomegalovirus genomes sequenced from clinical samples
Source: Virus Evol. 2022 Dec 5;8(2):veac114. doi: 10.1093/ve/veac114 (PMC10120596; doi:10.1093/ve/veac114)

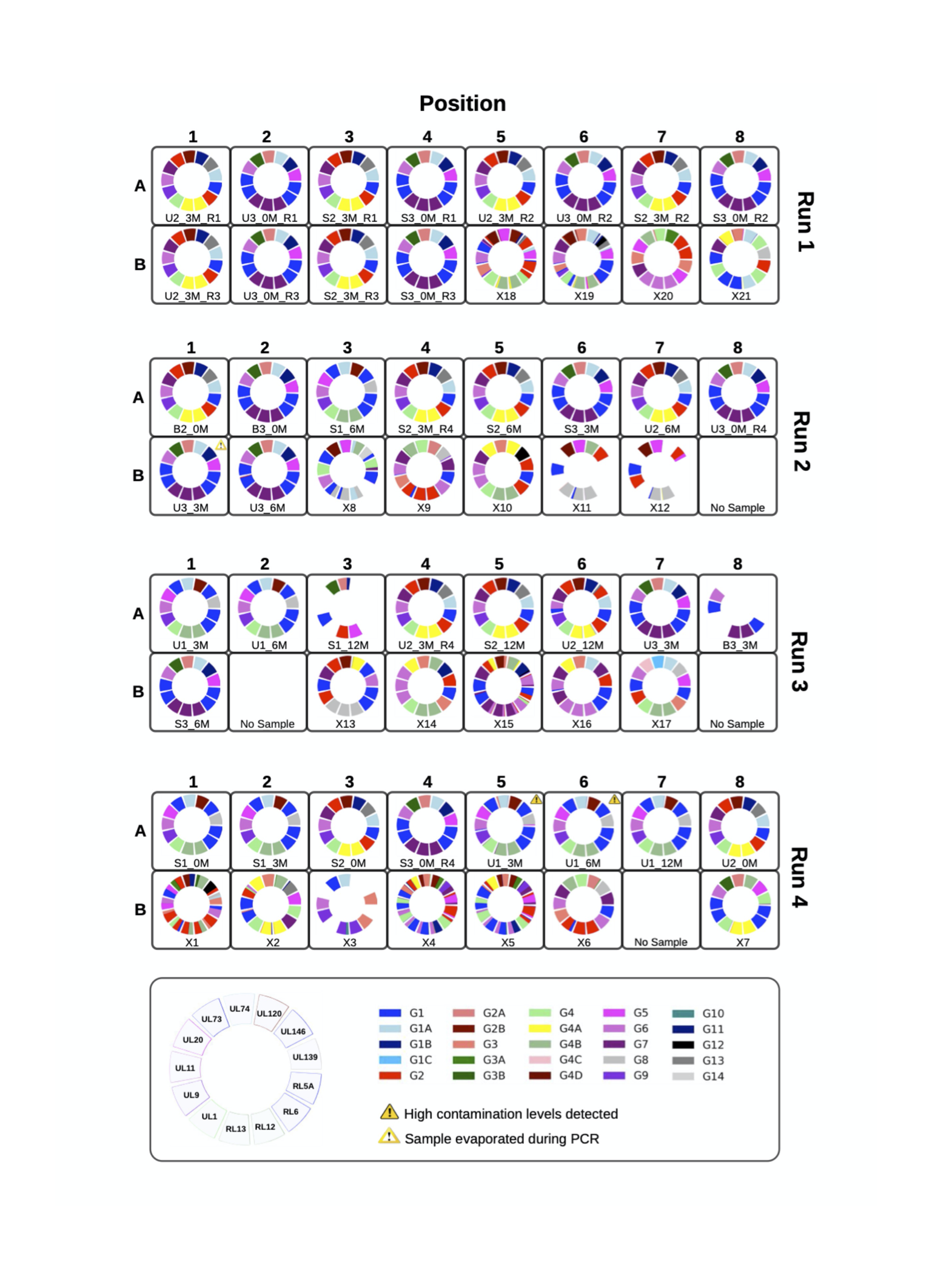

Supplement: veac114_Supp [file veac114_supp.zip › suppl_data/Figure S1.tiff]

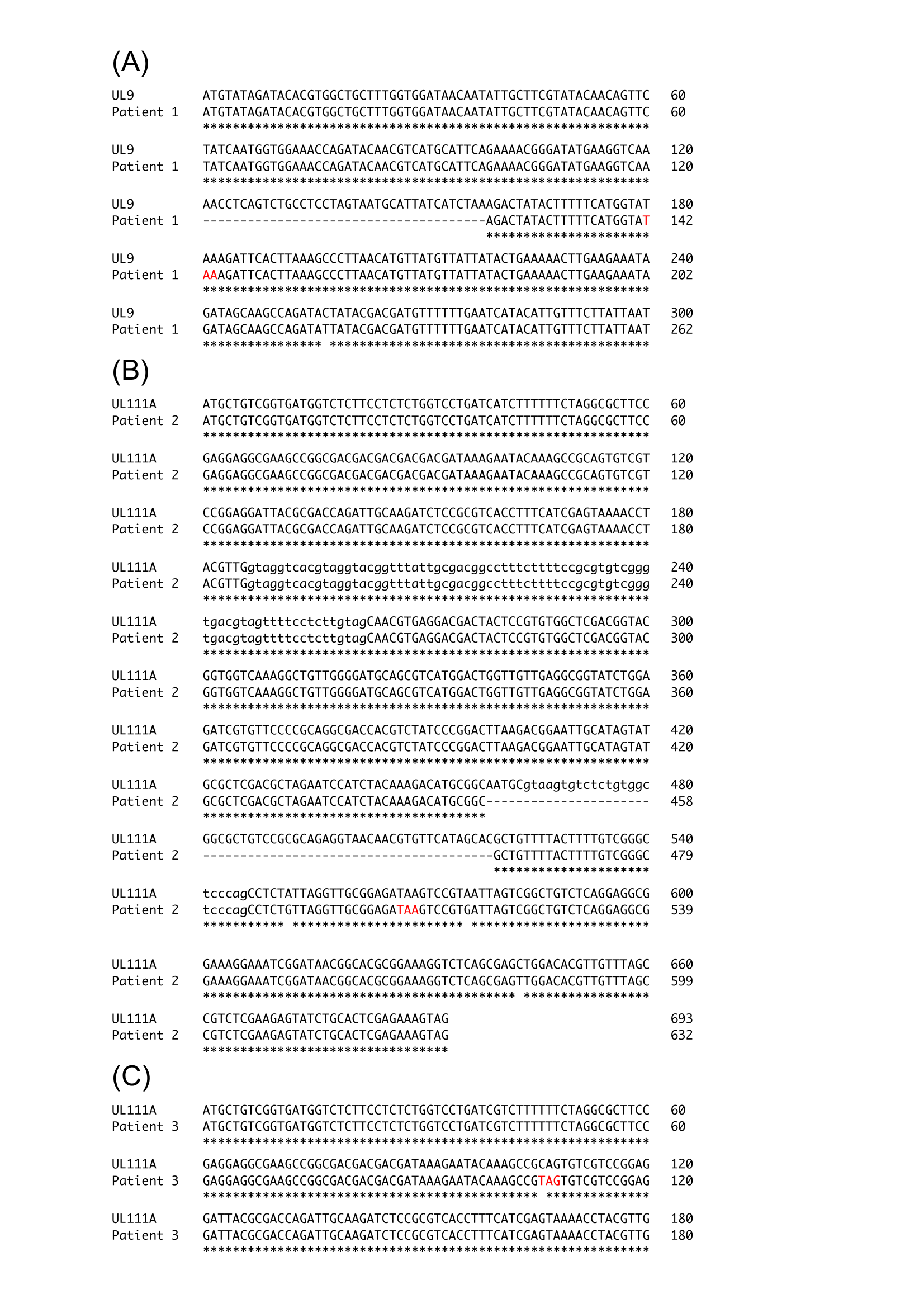

Supplement: veac114_Supp [file veac114_supp.zip › suppl_data/Figure S2 - Disrupted genes - HR.tiff]

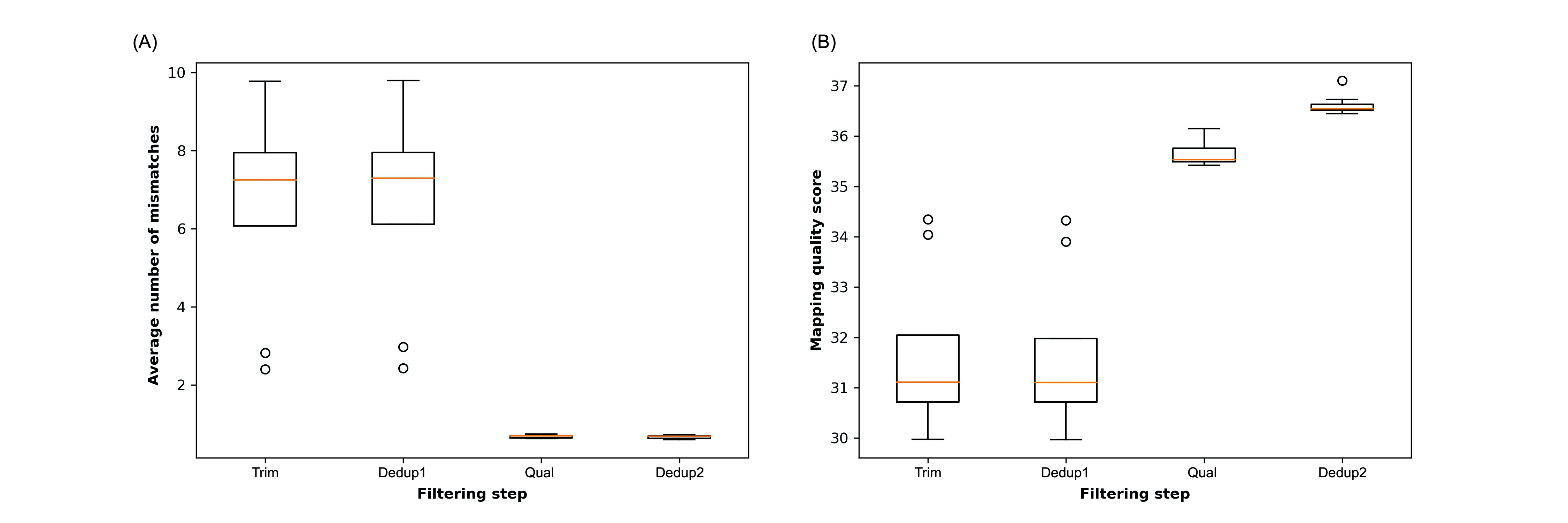

Supplement: veac114_Supp [file veac114_supp.zip › suppl_data/Figure S3 - Mapping scores.jpg]

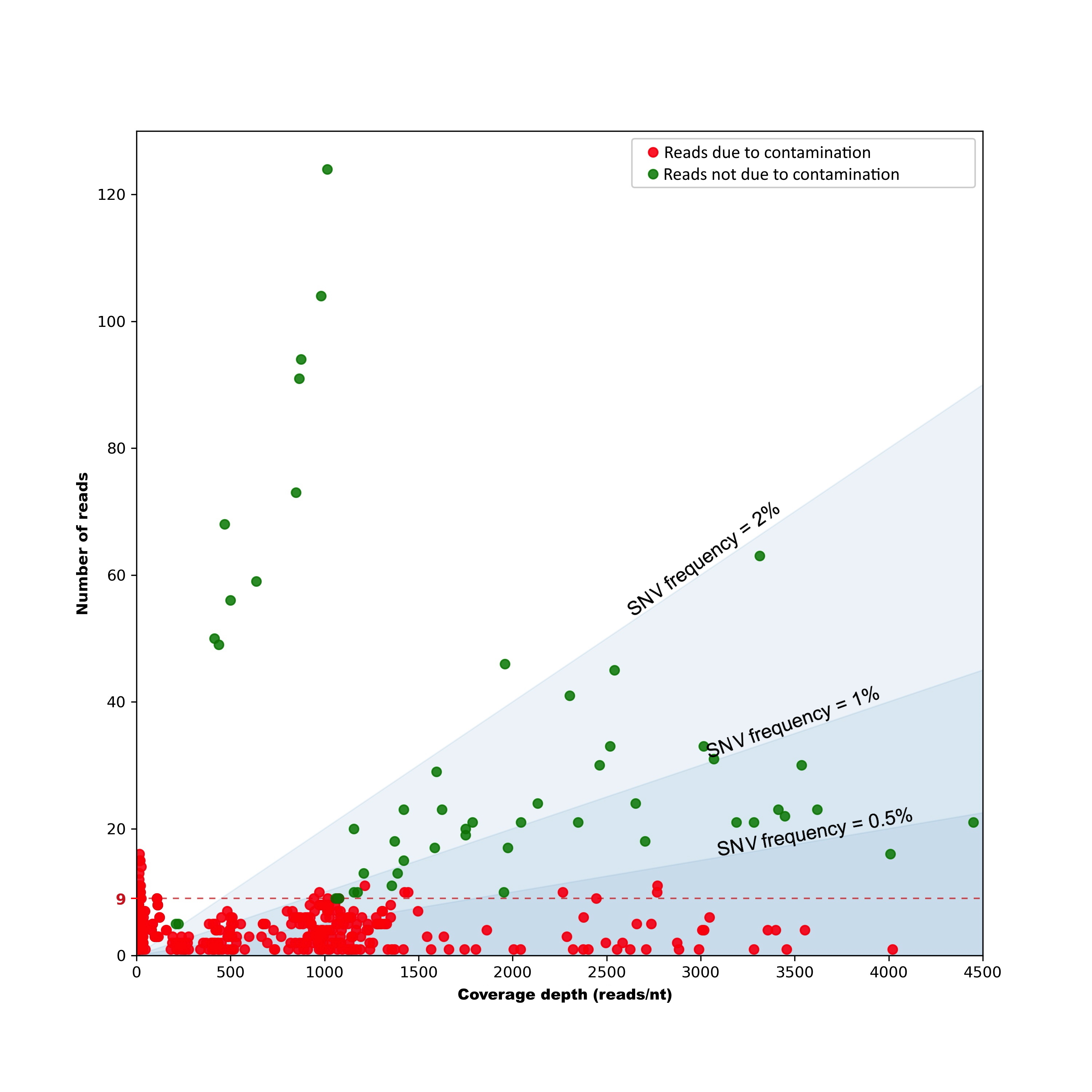

Supplement: veac114_Supp [file veac114_supp.zip › suppl_data/Figure S4 - SNV proportions.jpg]

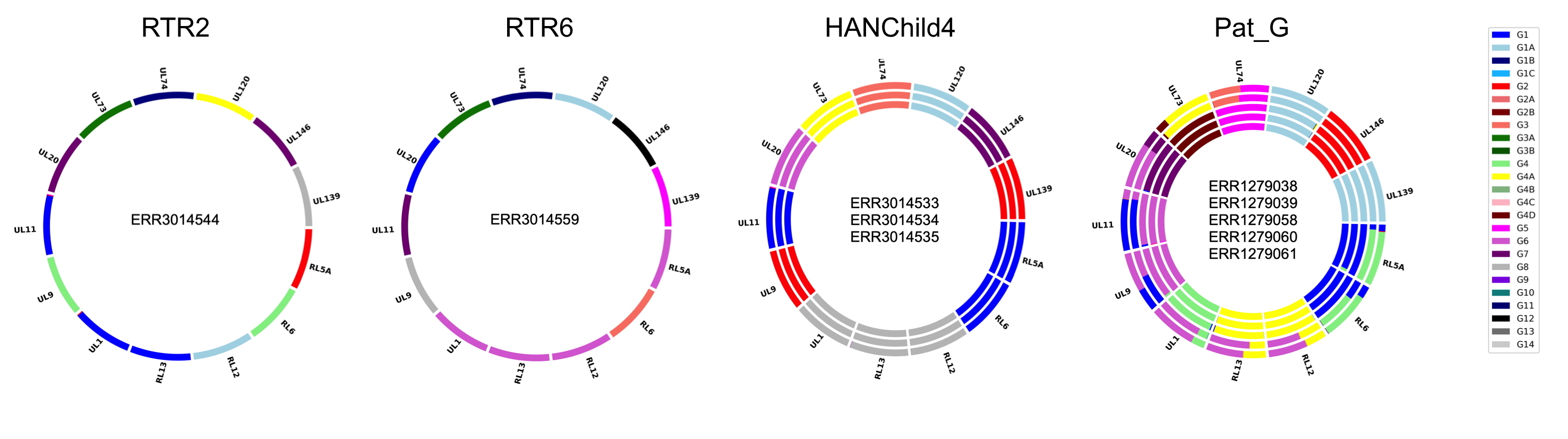

Supplement: veac114_Supp [file veac114_supp.zip › suppl_data/Figure S5 - Genotyping of public datasets.jpg]

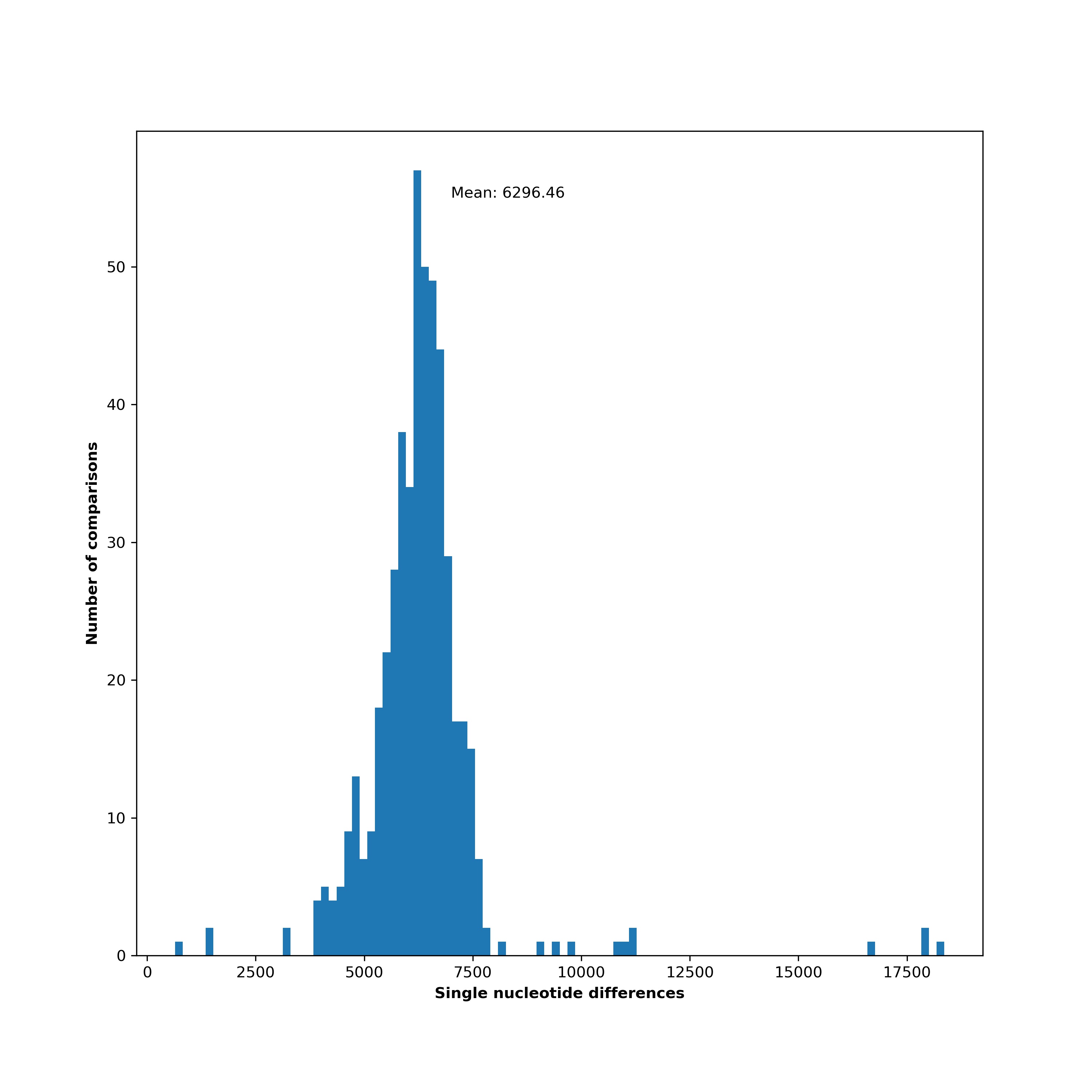

Supplement: veac114_Supp [file veac114_supp.zip › suppl_data/Figure S6 - Interstrain variability.jpg]

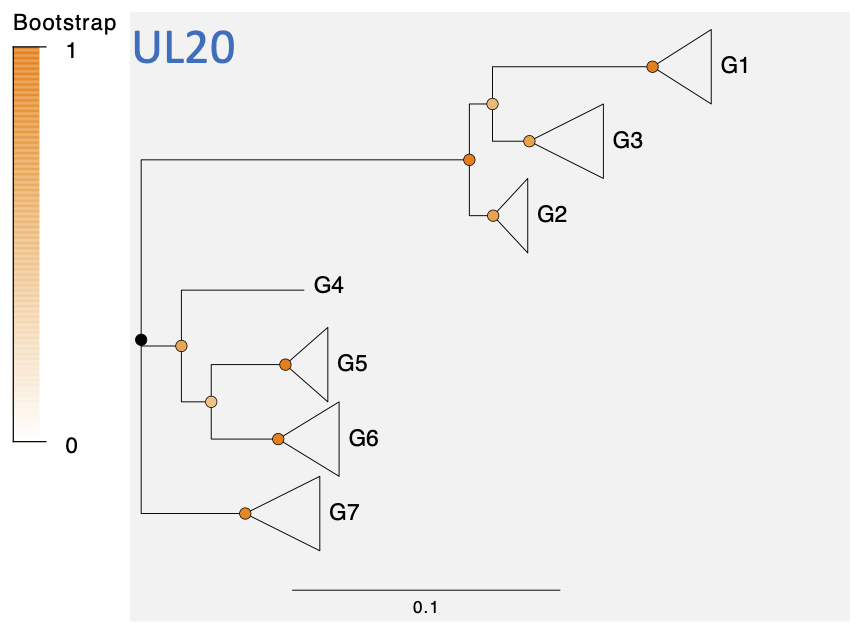

Supplement: veac114_Supp [file veac114_supp.zip › suppl_data/Figure S7 - UL20 tree - HR.tiff]
